# Severe Constipation in Parkinson's Disease and in Parkinsonisms: Prevalence and Affecting Factors

**DOI:** 10.3389/fneur.2019.00621

**Published:** 2019-06-18

**Authors:** Giuseppe Frazzitta, Davide Ferrazzoli, Annarita Folini, Grazia Palamara, Roberto Maestri

**Affiliations:** ^1^MIRT ParkProject, Livorno, Italy; ^2^Parkinson's Disease and Movement Disorders Department, “Moriggia-Pelascini” Hospital, Como, Italy; ^3^Istituti Clinici Scientifici Maugeri IRCCS, Department of Biomedical Engineering of the Montescano Institute, Pavia, Italy

**Keywords:** constipation, Parkinson's disease, Parkinsonisms, exercise, mobility

## Abstract

**Background:** constipation is one of the most common and disabling non-motor symptoms of Parkinson Disease (PD) and Parkinsonisms (PS). Few studies evaluate the difference of prevalence between PD and PS and the cause leading to a severe constipation in this diseases.

**Objective:** Aim of our study is to evaluate the prevalence of constipation in a population of patients with PD and PS and to evaluate which factors influence the development of severe constipation.

**Methods:** Two hundred and fifty outpatients with PD and 39 with PS were enrolled. Sixty five age-matched healthy subjects served as control. Constipation was assessed using the “Constipation Scoring System” (CSS). All patients underwent a global clinical, functional and neuropsychological assessment including: Unified Parkinson's disease Rating Scale (UPDRS), 6-min Walk Test (6MWT), and Mini-Mental State Examination (MMSE).

**Results:** Data confirm the high prevalence of constipation among patients with PD and PS. Severe constipation affects much more patients with PS. A significant association between total CSS and age, H and Y stage, 6MWT, MMSE, total UPDRS, and UPDRS III was found in PD. In PS patients total CSS was associated with age, 6MWT, total UPDRS, and UPDRS III. Multivariable regression analysis showed that the only variables significantly and independently associated with total CSS in PD patients were age and total UPDRS, both with direct relationship.

**Conclusions:** The reduction of motor performance seems to be the primary cause for developing severe constipation in PD and PS patients. These data suggest that maintain a good quality of gait and endurance may be helpful to reduce the risk of constipation.

## Introduction

Parkinson's disease (PD) and Parkinsonism (PS) are neurodegenerative disorders characterized by motor (rigidity, bradykinesia, and tremor) and non-motor symptoms (constipation, sleep disturbances, pain, depression). Non-motor symptoms characterize all phases of these diseases and have a relevant impact on the quality of life (QoL) of patients (1, 2). Nevertheless, these symptoms are often overlooked (3).

Constipation is one of the most common and disabling non-motor symptoms and is defined as an “unsatisfactory defecation characterized by infrequent stools, difficult stool passage, or both. Difficult stool passage includes straining, a sense of difficulty passing stool, incomplete evacuation, hard/lumpy stool, prolonged time to pass stool. Chronic constipation is defined as the presence of these symptoms for at least 3 months” (4). In the general population, constipation has a relevant impact on QoL (5) and increases the risk of intestinal obstruction, a well-known cause of reduced life expectancy (6). Moreover, the gastrointestinal dysfunctions may lead to heterogeneous absorption of L-dopa, which in turn contributes to motor fluctuations in PD.

The prevalence of constipation in PD has been reported with a wide spectrum ranging from 7 to 71% among different studies (7), mainly due to the different diagnostic criteria (8). Instead, only few studies addressed the topic of constipation in patients with PS (9).

A recent literature review shows that the median constipation prevalence in PD is about 40–50% and that constipation is related to disease duration (8).

Emerging evidence suggests that the neurodegenerative process in PD starts in the enteric nervous system and spreads via the vagus to the lower brainstem and the dopaminergic nigrostriatal system (10). This hypothesis could explain the pathogenesis of constipation and why this symptom precedes the development of PD (11). An involvement of autonomic regulatory structures has been also proposed as the pathological substrate for the autonomic dysfunction, including constipation, in patients with PS (12, 13).

Moreover, earlier onset of constipation has been associated with a more rapid disease progression and reduced survival in patients with PSP (14).

Given the dimension of the problem and its pathophysiologic and prognostic impact for both PD and PS, constipation remains an issue that has to be clarified and better characterized.

The aims of our study were (i) to evaluate the prevalence of constipation in an Italian population of PD patients and in a group of patients suffering from PS (Progressive Supranuclear Palsy—PSP, and Multisystem Atrophy—MSA), (ii) to compare prevalence data with those from a group of healthy controls, and (iii) to find relationships among the presence of constipation and clinical, functional and cognitive aspects of the disease.

## Methods

This was an observational, prospectic study. Between January and December 2018 we enrolled 250 outpatients with PD and 39 outpatients with PS (26 MSA and 13 PSP) at the Department of Parkinson's disease and Brain Injury rehabilitation of the “Moriggia-Pelascini” Hospital (Gravedona ed Uniti, Como-Italy). Inclusion criteria were: (i) diagnosis of idiopathic PD according to the UK Brain Bank criteria (15), (ii) Hoehn and Yahr (H&Y) stage 1–5 (for patients with PD), (iii) diagnosis of PSP or MSA (16, 17), (iv) stable pharmacological treatment both for Parkinson's disease and constipation during the last 4 weeks before the enrolment.

Exclusion criteria were: (i) structural gastrointestinal abnormalities (including abdominal mass, tumors, and colorectal polyposis) diagnosed with colonoscopy and/or barium enema, (ii) history of previous abdominal surgery, (iii) history of colorectal diseases, (iv) cardiovascular, endocrine, or neuromuscular diseases, (v) pharmacological treatments potentially affecting bowel motility and defecation (such as antidepressants, spasmolytics, or opioids).

We also included in this study 65 age-matched healthy subjects as controls. The study was approved by the local Scientific Committee and Institutional Review Board (Comitato Etico intera-aziendale delle province di Lecco-Como-Sondrio) and was in accordance with the code of Ethics of the World Medical Association (Declaration of Helsinki, 1967). A complete explanation of the study protocol was provided and written informed consent was obtained from all participants before their participation in the study.

We performed the evaluation of constipation using the Constipation Scoring System (CSS) (18). CSS is one of the most adopted tools for evaluating the prevalence and severity of constipation (19) and its use is widespread in Italy (20). Differently from the ROME III diagnostic criteria (21) the CSS permits to evaluate the severity of constipation. The questionnaire includes different variables: frequency of bowel movement, difficulty (painful evacuation effort), completeness (feeling incomplete evacuation), abdominal pain, time (minutes in lavatory per attempt), type of assistance for defecation, failure (unsuccessful attempts for evacuation per 24 h), and history (duration of constipation). A scoring range from 0 (normal) to 4 (severe condition) (with the exception of assistance for defecation, ranging from 0 to 2) is derived. A global score is obtained by adding each individual score. Finally, constipation is graduated as mild (score 1–5), moderate (6–10), severe (11–15), and very severe (15–30) (22). CSS questions are very simple and the questionnaire can be completed in about 5 min by the patients. A nurse was always present and available to help patients in case of doubts.

All patients underwent a global clinical, functional, and neuropsychological assessment including: Unified Parkinson's disease Rating Scale (UPDRS), 6-min Walk Test (6MWT), and Mini-Mental State Examination (MMSE). The reliability and applicability of UPDRS for patients suffering from PSP and MSA was verified (23, 24).

All the assessments were carried out in the same day.

## Statistical Analysis

The central tendency and the dispersion of continuous variables are reported as mean ± SD. Descriptive statistics for categorical variables are reported as N (percent frequency).

Between-group comparisons of PD patients, patients with PS and controls for continuous variables were carried out by one-way ANOVA. Following a significant result for ANOVA, *post-hoc* analyses were performed to compare pairwise differences in groups. The Tukey-Kramer adjustment for multiple comparisons was used. Adjusted *p*-values were reported when appropriate.

Between-group comparisons of PD patients and patients with PS were carried out by unpaired *t*-test.

The association between the severity of constipation (absence, mild, moderate, severe, and very severe) and each group of subjects was investigated by contingency tables. Comparisons of categorical variables were carried out by the Chi-square test.

The association between couples of variables was assessed by the Pearson correlation coefficient.

Multivariable regression methods were used to assess the relationship between constipation (CSS global score) and explanatory variables (age, disease duration, l-dopa eq dosage, 6MWT, MMSE, and total UPDRS). Finally, the likelihood of having either moderate, severe, or very severe constipation vs. absence of constipation or mild constipation as a function of potential predictors was assessed by logistic regression.

All statistical tests were two-tailed and statistical significance was set at *p* < 0.05. All analyses were carried out using the SAS/STAT statistical package, release 9.4 (SAS Institute Inc., Cary, NC, U.S.A.).

## Results

Considering the two types of patients included in the PS group, namely PS-MSA (*N* = 26) and PS-PSP (*N* = 13), despite potentially affected by autonomic dysfunction to a different extent, no differences were observed in all considered demographic, clinical and cognitive variables (Table 1) and in global CSS values (6.04 ± 4.38 vs. 5.54 ± 4.54, *p* = 0.66) as well as in the value of all its items (all *p* > 0.30). Hence, in all subsequent analyses, PS patients were considered as a single population. The demographic, clinical, and cognitive characteristics of PD and PS patients are reported in Table 2. No difference in age, gender, and cognitive state were observed. Disease duration, functional capacity, as assessed by the 6MWT, and L-dopa eq dosage were significantly different in the two groups, as expected. Healthy controls (*N* = 65, 34% males) had similar age (67.0 ± 7.5 years, *p* > 0.25 for the comparison with both groups of patients).

**Table 1:** Demographic, clinical, and cognitive characteristics of PS-MSA and PS-PSP patients.

**Variable**	**PS-MSA patients (*N* = 26)**	**PS-PSP patients (*N* = 13)**	***p*-value**
Age (years)	68.6 ± 10.4	71.8 ± 6.3	0.42
Gender (% of males)	13 (50%)	5 (38%)	0.49
Disease duration (years)	6.2 ± 3.9	5.3 ± 3.1	0.56
l-dopa eq (mg/day)	519 ± 387	313 ± 188	0.12
6MWT(m)	218 ± 118	192 ± 120	0.60
UPDRS III	24.7 ± 8.5	27.0 ± 5.4	0.44
Total UPDRS	52.1 ± 17.6	55.6 ± 9.0	0.47
MMSE	25.9 ± 3.7	27.0 ± 2.0	0.36

*PD, Parkinson's disease; PS, Parkinsonian syndromes; l-dopa eq, levodopa equivalent dose; mg, milligrams; 6MWT, 6-min walk test; UPDRS, Unified Parkinson's disease Rating Scale; MMSE, Mini Mental State Examination*.

**Table 2:** Demographic, clinical, and cognitive characteristics of PD and PS patients.

**Variable**	**Patients with PD (*N* = 250)**	**Patients with PS (*N* = 39)**	***p*-value**
Age (years)	68.4 ± 8.9	69.7 ± 9.2	0.28
Gender (% of males)	143 (57.2%)	18 (46.2%)	0.20
Disease duration (years)	9.9 ± 5.2	5.9 ± 3.7	<0.0001
l-dopa eq (mg/day)	688 ± 358	458 ± 351	0.0006
6MWT (m)	290 ± 135	209 ± 118	0.0006
UPDRS III	21.6 ± 6.3	25.3 ± 7.8	0.001
Total UPDRS	44.7 ± 13.7	53.1 ± 15.7	0.0007
MMSE	26.3 ± 3.4	26.2 ± 3.3	0.87

*PD, Parkinson's disease; PS, Parkinsonian syndromes; l-dopa eq, levodopa equivalent dose; mg, milligrams; 6MWT, 6-min walk test; UPDRS, Unified Parkinson's disease Rating Scale; MMSE, Mini Mental State Examination*.

The contingency table for the association between constipation severity and groups is shown in Table 3: 80, 92, and 55% of PD patients, PS patients and healthy controls, respectively, suffered from some level of constipation (*p* < 0.0001 for between group comparison).

**Table 3:** Contingency for the association between constipation severity (according to the Constipation Scoring System) and groups.

**Variable**	**No constipation**	**Mild constipation**	**Moderate constipation**	**Severe constipation**	**Very severe constipation**
Patients with PD (%)	51 (20.4%)	106 (42.4%)	76 (30.4%)	15 (6.0%)	2 (0.8%)
Patients with PS (PS-MSA+PS-PSP, %)	3 (2+1, 7.7%)	19 (12+7, 48.7%)	9 (7+2, 23.1%)	8 (5+3, 20.5%)	0 (0.0%)
Healthy controls (%)	29 (44.6%)	32 (49.2%)	2 (3.1%)	2 (3.1%)	0 (0.0%)

*p <0.0001 for between group comparison (see the text)*.

*PD, Parkinson's disease; PS, Parkinsonian syndromes*.

The global CSS values as well as the values of all its items are given in Table 4 for the three groups of subjects considered. For all items, except the two assessing pain, ANOVA revealed a highly significant difference in the three groups. *Post hoc* analysis revealed that no differences were found between PD and PS patients and that both groups significantly differed from healthy controls.

**Table 4:** CSS values (global score and each item score) for patients with PD, patients with PS, and healthy controls.

**Variable**	**Patients with PD**	**Patients with PS**	**Healthy controls**	***p*-value**
CSS Global Score	4.39 ± 3.78	5.87 ± 4.38	1.58 ± 2.83^‡^	<0.0001
Frequency of bowel movement	0.40 ± 0.58	0.51 ± 0.60	0.09 ± 0.29^†^	<0.0001
History (duration of constipation)	1.06 ± 1.32	1.46 ± 1.59	0.29 ± 0.86^‡^	<0.0001
Difficulty (painful evacuation effort)	0.26 ± 0.72	0.38 ± 0.75	0.23 ± 0.61	0.52
Completeness (feeling incomplete evacuation)	0.76 ± 1.00	1.08 ± 1.20	0.29 ± 0.65^†^	0.0001
Abdominal pain	0.30 ± 0.67	0.33 ± 0.81	0.23 ± 0.49	0.69
Type of assistance for defecation	0.42 ± 0.61	0.62 ± 0.75	0.09 ± 0.29^†^	<0.0001
Time (minutes in lavatory per attempt)	0.71 ± 0.84	0.97 ± 0.63	0.29 ± 0.68^†^	<0.0001
Failure (unsuccessful attempts for evacuation per 24 h)	0.47 ± 0.68	0.51 ± 0.64	0.06 ± 0.24^‡^	<0.0001

‡ *p <0.0001 for the comparison with PD patients and patients with PS*.

† *p <0.001 for the comparison with PD patients and patients with PS*.

*CSS, Constipation Scoring System; PD, Parkinson's disease; PS, Parkinsonian syndromes*.

Correlation analysis in PD patients showed a significant association between total CSS and age (*r* = 0.27, *p* < 0.0001), H&Y stage (*r* = 0.25, *p* = 0.0001), 6MWT (*r* = −0.22, *p* = 0.0006), MMSE (*r* = −0.16, *p* = 0.013), total UPDRS (*r* = 0.27, *p* < 0.0001), UPDRS III (*r* = 0.27, *p* < 0.0001), UPDRS II (*r* = 0.28, *p* < 0.0001), and UPDRS I (*r* = 0.18, *p* = 0.005).

The same analysis carried out in PS patients revealed that total CSS was associated with age (*r* = 0.31, *p* = 0.05), 6MWT (*r* = −0.47, *p* = 0.004), total UPDRS (*r* = 0.47, *p* = 0.004), UPDRS III (*r* = 0.42, *p* = 0.01), and UPDRS II (*r* = 0.42, *p* = 0.01).

Multivariable regression analysis showed that, out of all potential predictors considered, the only variables significantly and independently associated with total CSS in PD patients were age (beta = 0.093, *p* = 0.001) and total UPDRS (beta = 0.060, *p* = 0.022), both with direct relationship (overall *R*^2^ = 0.2).

Logistic regression analysis (Table 5) confirmed that only age and total UPDRS were significant and independent predictors of the occurrence of moderate, severe and very severe constipation. Odds ratio indicated that a 1-year increase in age or 1-point increase in total UPDRS were associated, respectively, with a 6% and nearly 4% increase in the risk of having moderate-severe-very severe constipation.

**Table 5:** Logistic regression analysis (see the text, results section).

**Variable**	**Estimate**	***p*-value**	**Point estimate**	**95% low**	**95% high**
Age (years)	0.058938	0.003	1.061	1.020	1.103
Gender	−0.65527	0.054	0.519	0.267	1.011
Side of motor symptoms predominance	−0.28617	0.36	0.751	0.409	1.378
Disease duration (years)	0.022316	0.48	1.023	0.961	1.089
6MWT	0.0010831	0.50	1.001	0.998	1.004
l-dopa eq (mg/day)	6.1148e-05	0.90	1.000	0.999	1.001
MMSE	−0.094335	0.08	0.910	0.820	1.010
Total UPDRS	0.037473	0.024	1.038	1.005	1.073

*6MWT, 6-min walk test; l-dopa eq, levodopa equivalent dose; mg, milligrams; MMSE, Mini Mental State Examination; UPDRS, Unified Parkinson's disease Rating Scale*.

In PS patients, multivariable regression analysis showed that none of potential predictors considered was independently associated with total CSS, with a borderline value only for total UPDRS (*p* = 0.06).

## Discussion

The study confirms the high prevalence of constipation among patients with PD or PS, with a more relevant incidence of severe form of constipation in PS (20 vs. 7% in PD).

We did not find a correlation among disease duration, l-dopa eq dosage, and total CSS for patients with PD or PS. These data support the hypothesis that the neuropathological involvement of the gastrointestinal system in PD patients precedes the onset of motor symptoms (25) and confirm that people with constipation may have a relatively high risk of developing PD (26). The lack of a correlation between L-dopa eq dosage and total CSS is worthy of consideration as some authors have previously attributed constipation to dopaminergic therapy (27). These results also confirm that PS patients present no drug-responsive symptoms of autonomic dysfunctions, regardless the disease duration.

Correlation analysis showed that the severity of constipation in both PD and PS patients was strongly, negatively associated with the 6MWT: the less the patients walked, the more they were constipated.

This evidence is clinically relevant: patients with movement disorders have to be trained to walk in order to reduce the risk of presenting a severe constipation. It is widely known that the regular exercise reduce the risk of constipation, but this study demonstrate the relevance to maintain a good walking endurance to prevent a severe constipation in patients with PD and PS.

Our results showed that only age and total UPDRS were significant and independent predictors of the occurrence of moderate-severe and very severe constipation in patients with PD. This observation is in line with previous findings suggesting that the severity and the prevalence of constipation increase with age (change in life style, medications, underlying diseases, rectal sensory-motor dysfunction) (28). Moreover, these data demonstrate that the worsening of the clinical conditions (evaluated with total UPDRS) is detrimental for the gastrointestinal functioning in people with PD, as the reduction of motor performance seems to worsen constipation.

PD is a combination of motor and non-motor symptoms. Among non-motor symptoms, constipation is one of the more relevant and difficult to treat. It is known that the dopaminergic therapy does not improve neither bowel frequency nor defecating difficulties (29): results from this study confirm this observation and show that constipation does not get worse increasing the dopaminergic therapy.

The motor disturbances, particularly gait and balance dysfunctions, have a relevant impact on patients' conditions leading to reduced autonomy in activities of daily living. As well as it happens for the non-motor disturbances (as constipation), the pharmacological therapy and the surgical treatments (deep brain stimulation) do not significantly improve gait and balance dysfunctions (30, 31). Nevertheless, different recently described rehabilitative treatment have showed to be effective on gait and balance, both in PD and PS, leading in turn to an improvement in autonomy in the activities of daily living and QoL (32–38). Interestingly, in several studies, it has been showed that an intensive and regular exercise improves properly the performance on the 6MWT (33, 35, 36).

This is a relevant point, as the worsening of constipation in this study was related with poorer performances (meters walked) in the 6MWT and worst scores in total UPDRS. Therefore, it is possible to assume that doing exercises from the time of diagnosis may be important to maintain a good motor activity and avoid the development or the worsening of constipation. Not least, patients must observe the rules of an adequate nutrition and hydration to facilitate the intestinal activity.

As previously mentioned, also in patients with PS there is a high incidence of constipation and, its severity was strictly related to the worsening in the 6MWT performance. Patients with PS suffer from autonomic dysfunctions whose severity is much more pronounced than in PD. This evidence could explain the higher incidence of constipation in PS (92.3% in PS vs. 79.6% in PD) and the rate of severe form in this population (20.5% in PD vs. 6.8% in PS). Nevertheless, it is unknown whether symptoms such as constipation in PS are the result of dysautonomia due to direct involvement by the neurodegenerative process or secondary to the combination with other external factors (14).

In conclusion, we found that the reduction of motor performance seems to contribute to the development of a severe constipation. Therefore, our data testify that the degree of mobility plays a relevant deterministic pathophysiological role in the development of constipation, suggesting that the improvement of gait capacity and endurance could be helpful for reducing the risk of constipation.

## Study limitations

This study has some limitations:

- We have considered PD patients with different H-Y stages and this can be a confounding factor in the comparison between patients with PD and patients with PS. As a matter of fact, the vast majority of considered PD patients (>96%) was in H-Y stages 2–4, providing a realistic picture of the clinical conditions of PD patients. Moreover, constipation is a symptom that may precede the onset of motor symptoms and diagnosis by many years.
- For the evaluation of the cognitive function, we have used the MMSE, which is not a specific tool for PD patients. However, the aim of our paper was not to evaluate the different and well know cognitive aspects of PD and PS, but only to provide a global evaluation of cognitive function in the considered populations.
- Constipation was assessed using the CSS. Even though CSS is one of the most adopted tools for evaluating the prevalence and severity of constipation, especially in Italy, it has not been validated in patients with PD or Parkinsonism. However, we carried out correlation analysis (Spearman r) to assess the association between CSS total score and the item 1.11 of MDS-UPDRS, and found a highly significant, very strong association (*r* = 0.92, *p* < 0.0001).

## Data Availability

The datasets for this manuscript are not publicly available because the raw data supporting the conclusions of this manuscript will be made available by the authors, without undue reservation, to any qualified researcher. Requests to access the datasets should be directed to frazzittag62@gmail.com.

## Ethics Statement

The study was approved by the local Scientific Committee and Institutional Review Board (Comitato Etico intera-aziendale delle province di Lecco-Como-Sondrio) and was in accordance with the code of Ethics of the World Medical Association (Declaration of Helsinki, 1967). A complete explanation of the study protocol was provided and written informed consent was obtained from all participants before their participation in the study.

## Author Contributions

GF, RM, AF, and GP contributed conception and design of the study and organized the database. RM performed the statistical analysis. GF, RM, and DF wrote the first draft of the manuscript and wrote sections of the manuscript. All authors contributed to manuscript revision, read and approved the submitted version.

### Conflict of Interest Statement

The authors declare that the research was conducted in the absence of any commercial or financial relationships that could be construed as a potential conflict of interest.
